# Computational fluid dynamics modelling in cardiovascular medicine

**DOI:** 10.1136/heartjnl-2015-308044

**Published:** 2015-10-28

**Authors:** Paul D Morris, Andrew Narracott, Hendrik von Tengg-Kobligk, Daniel Alejandro Silva Soto, Sarah Hsiao, Angela Lungu, Paul Evans, Neil W Bressloff, Patricia V Lawford, D Rodney Hose, Julian P Gunn

**Affiliations:** 1 Department of Cardiovascular Science, University of Sheffield, Sheffield, UK; 2 Insigneo Institute for In Silico Medicine, Sheffield, UK; 3 Department of Cardiology, Sheffield Teaching Hospitals NHS Trust, Sheffield, UK; 4 University Institute for Diagnostic, Interventional and Pediatric Radiology, University Hospital of Bern, Inselspital, Bern, Switzerland; 5 Faculty of Engineering & the Environment, University of Southampton, Southampton, UK

## Abstract

This paper reviews the methods, benefits and challenges associated with the adoption and translation of computational fluid dynamics (CFD) modelling within cardiovascular medicine. CFD, a specialist area of mathematics and a branch of fluid mechanics, is used routinely in a diverse range of safety-critical engineering systems, which increasingly is being applied to the cardiovascular system. By facilitating rapid, economical, low-risk prototyping, CFD modelling has already revolutionised research and development of devices such as stents, valve prostheses, and ventricular assist devices. Combined with cardiovascular imaging, CFD simulation enables detailed characterisation of complex physiological pressure and flow fields and the computation of metrics which cannot be directly measured, for example, wall shear stress. CFD models are now being translated into clinical tools for physicians to use across the spectrum of coronary, valvular, congenital, myocardial and peripheral vascular diseases. CFD modelling is apposite for minimally-invasive patient assessment. Patient-specific (incorporating data unique to the individual) and multi-scale (combining models of different length- and time-scales) modelling enables individualised risk prediction and virtual treatment planning. This represents a significant departure from traditional dependence upon registry-based, population-averaged data. Model integration is progressively moving towards ‘digital patient’ or ‘virtual physiological human’ representations. When combined with population-scale numerical models, these models have the potential to reduce the cost, time and risk associated with clinical trials. The adoption of CFD modelling signals a new era in cardiovascular medicine. While potentially highly beneficial, a number of academic and commercial groups are addressing the associated methodological, regulatory, education- and service-related challenges.

## Introduction

Computational fluid dynamics (CFD) is a well-established tool used in engineering, in many areas of which it has become the primary method for design and analysis. Bioengineers have adopted CFD to study complex physiological flows and have demonstrated their potential.^1^ There is increasing interest in applying these methods in cardiovascular medicine.^2^ ^3^ CFD-based techniques are being used to build complex computer representations (in silico models) of the cardiovascular system in health and disease. CFD modelling is a new field within cardiovascular medicine, enhancing diagnostic assessment, device design and clinical trials. It can predict physiological responses to intervention and compute previously unmeasurable haemodynamic parameters.^4^ As CFD modelling continues to translate into clinical tools, it is important that clinicians understand the principles, benefits and limitations of these techniques. This article explores these topics using state-of-the-art examples in key clinical areas, highlighting applications likely to impact clinical practice within the next 5 years (table 1).

**Table 1:** Summary of CFD modelling applications in cardiovascular medicine

Area	Clinical applications	Data and evidence	Potential clinical impact	Limitations and challenges	References
Coronary artery disease and physiology	Models based upon coronary angiography (CT /invasive) to compute physiological coronary lesion significance less invasively	Multiple trials demonstrating broadly good agreement between standard and CFD-derived FFR (vFFR). Lesion significance established in ∼80–90%	Widened access to the benefits of physiological lesion assessment; vFFR lacks the practical limitations that restrict use of the invasive technique. Virtual stenting enables planning and selection of optimal treatment strategy	Accurate vessel reconstruction and patient-specific tuning of the model boundary conditions (especially those of myocardial resistance)	5 6 7 8
Valve prostheses	Evaluation and optimisation of prosthetic valve design from a haemodynamic perspective	Included in the design dossier given to RA for approval before use in humans. Third party, comparative studies are in engineering literature	CFD modelling enables the best design, yielding the optimal haemodynamics and lowest achievable risk of design-related thrombosis and thromboembolism	Dependence upon validity of models to interpret fluid stresses in terms of thrombogenic/haemolytic potential. Primarily relates to mechanical valves. Tissue valve leaflets remain challenging to model	9 10 11
Native valve haemodynamics in health and disease	Non-invasive computation and quantification of trans-valvular pressure drop and regurgitant fraction from CT imaging	Accurate 3D simulations in patient-specific models with valves in open and closed states to predict transvalvular dynamics in diseased states	Improved objective assessment and surveillance of valve disease from non-invasive imaging data	Requires high quality 3D images of valve orifice—not routinely generated. Balancing the requirement for complex dynamic simulation (FSI) vs simpler models (valve open/closed)	12 13
Aortic aneurysm	Provides quantitative haemodynamic data for non-invasive imaging to emphasise the significance of findings. Virtual therapy simulation/predictions	No published outcome trials, only single centre experiences and small cohorts using different boundary condition and computational methods	To better predict aneurysm progression and risk of rupture. Prediction of putative therapeutic effects. Individualised care and reduction in costs for unnecessary follow-up imaging and visits	Impact of low image contrast structures of aortic aneurysm (eg, wall, thrombus) as well as wall motion needs to be further assessed. CFD alone is probably limited and needs to be complemented by, for example, FSI	3 14 15 16
Aortic dissection	Pathophysiological conditions in true and false lumen computed from non-invasive boundary conditions (CT and MRI+PC). Effects of virtual therapy.	No published outcome trials, only single centre experiences and small cohorts using different boundary conditions and computational methods	Computed pressure and flow conditions used to guide (semi-) invasive therapeutic procedure decisions. Physiological effects of therapies can be simulated and better predicted	Significant early and late re-modelling of the dissected wall. Entry, re-entry and communication channels create a complex computational scenario. CFD alone might be limited. A potential role for FSI	17 18 19
Stent design	Prediction of WSS and related metrics that influence endothelial function and NH due to stent-induced haemodynamic disturbance	Turbulent or disturbed laminar flow reduces WSS stimulating adverse vessel remodelling. NH preferentially accumulates in these regions	Not possible to measure arterial WSS in vivo, especially in the vicinity of stent struts post-PCI. Modelling provides detailed analysis of flow, and the influence of stent design through patient-specific reconstructions, enabling the optimal stent design to be achieved	High resolution imaging, vessel reconstruction and boundary conditions are challenging. CFD simulations demand fine computational meshes and time-resolved pulsatility. Run-times are long, even with high performance computing	20 21 22 23 24 25 26 27
Cerebral aneurysm	Prediction of intra-aneurysmal flow, stasis, jet impingement and WSS from MRI and CT cerebral angiography data	Published data on association between WSS, aneurysm initiation, growth, and potentially rupture	Detailed, individualised haemodynamic analysis with potential for risk prediction. Impact of putative treatments on local haemodynamics evaluated in silico	Difficulty interpreting complex and detailed WSS results. Understanding how results translate to rupture risk. Validation of rupture predictions—a rare event	4 28 29
Pulmonary hypertension (PH)	Greater insights into complex PH physiology. Increasing interest in non-invasive diagnosis and monitoring of response to treatment	Models based on MR flows demonstrated to differentiate between healthy volunteers and to stratify PH subcategories	Imaging-based modelling of pulmonary haemodynamics can reduce the requirement for right heart catheterisation. Models show association between reduced WSS and invasive PH metrics. PH subtype characteristics simulated to understand the structural changes contributing to increased PAP	Spatial resolution of imaging and segmentation protocols. The use of a pressure surrogate measure. The presence of many outlets requiring many measurements to tune the outflow boundary conditions	30 31 32 33
Arterial wall shear stress (WSS)	WSS mapping, cross-referenced with vascular disease phenotype, is contributing to the understanding of cellular biology	An abnormal WSS pattern has been correlated with vascular diseases, including atherosclerosis, aneurysm and post-stent NH	Ultimate understanding of the development and progression of atherosclerosis. WSS map combined with multi-scale modelling may inform clinical practice, such as the site of rupture in aneurysm, and severity of in-stent restenosis.	A detailed vascular geometry is essential for an accurate WSS map. Acquisition of patient specific boundary conditions remains clinically challenging.	20 21 22 25
Heart failure	Models based upon CT and MR help compute haemodynamics and the spatio-temporal distributions of pressure and myocardial stress/strain	CFD/FSI models replicate realistic pathophysiology in models of health and disease (eg, HFREF, HFPEF, HCM, DCM, and RWMA post-MI)	Additional haemodynamic data potentially enables early diagnosis and stratifies disease phenotypes and severities. Characterising complex vortex flows identifies areas of flow stagnation and thrombus risk	Resolution of imaging and reconstruction (representing trabeculae and papillary muscles). Tuning with realistic boundary conditions. Requirement for FSI in many models	2 34 35 36 37 38 39 40 41
CRT	Coupled electro-mechanical models of the ventricle incorporating CFD (multi-physics models) used to investigate heart function	Published reports of accurate patient-specific haemodynamic simulations with sufficient detail to optimise CRT before surgical intervention	Improved selection of CRT responders. Simulation and selection of optimal tuning of device settings and lead placement on an individual case basis	Uncertainties and assumptions regarding boundary conditions and the range of clinical measurements required for parameterisation. Mesh generation, prolonged computation times	
VADs	Generic optimisation of pump design. Patient-specific models can aid implantation strategy and tuning of output according to patient physiology	Published models describing haemodynamic influences of catheter placement and minimisation of adverse haemodynamic effects	Pump tuning to ensure periodic opening and closing of AV, preventing leaflet fusion. Personalised catheter placement planning (prediction and avoidance stasis and thrombus formation)	Post-implantation imaging artefact limits modelling. Optimising performance requires the balance of multiple competing factors. As for all cardiac electromechanical models, selection of appropriate patient specific parameters is difficult due to sparsity of data	
Congenital heart disease	CFD simulates haemodynamics which are complex and hard to predict in the context of a diverse and heterogeneous range of disease phenotypes	Range of models described, including reduced order, 3D CFD, FSI and multiscale, particularly in the context of univentricular circulation, aortic and pulmonary malformations	Modelling enables greater understanding of systemic and regional haemodynamics and the prediction of response to putative surgical or device-based treatments which often involve significant modifications to the circulatory tree	Acquisition and application of model parameters and boundary conditions from patient and literature data. The ultimate personalisation challenge	42 43 44

AV, aortic valve; CFD, computational fluid dynamics; CRT, cardiac resynchronisation therapy; CT (A), CT (angiography); DCM, dilated cardiomyopathy; FSI, fluid solid interaction; HCM, hypertrophic cardiomyopathy; HFPEF, heart failure with preserved EF; HFREF, heart failure with reduced EF; MI, myocardial infarction; NH, neointimal hyperplasia; PAP, pulmonary artery pressure; PC, phase-contrast; PCI, percutaneous coronary intervention; RA, regulatory authority; RWMA, regional wall motion abnormality; (v)FFR; (virtual) fractional flow reserve; VAD, ventricular assist device; WSS, wall shear stress.

## What is CFD?

CFD is a specialist area of mathematics and a branch of fluid mechanics. It is used in the design of many safety-critical systems, including aircraft and vehicles, by solving differential equations to simulate fluid flow. A glossary of uesful terms is provided in table 2.

**Table 2:** CFD—a glossary of selected useful terminology

Analytical solution	Relatively simple models can be described by solving a number of equations using mathematical analysis techniques such as calculus or trigonometry. The solution is analytical because an exact solution can be obtained through algebraic manipulation of the equations (*cf.* numerical solution).
Bernoulli equation: 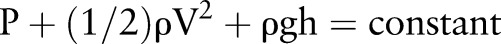 Rearranged and simplified: 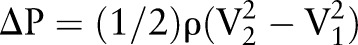	The Bernoulli equation relates blood pressure (*P*) and flow velocity (*V*). The total energy of flowing blood comprises hydrostatic (P), kinetic ((1/2)ρV^2^) and potential (ρgh where ρ is fluid density, g is gravity and h is height) energies, the sum of which is conserved. Therefore, an increase in flow velocity must be accompanied by a decrease in pressure, and vice versa. Gravitational effects (ρgh) are usually neglected in a supine vessel. The simplified and rearranged equation is used routinely to calculate transvalvular pressure gradients from flow velocity. The Bernoulli equation ignores energy loss due to viscous friction (see Poiseuille equation) and turbulence, and assumes steady flow.
Boundary conditions	A set of parameters or relationships which describe the physiological conditions (haemodynamic or structural) acting at the boundaries of a modelled segment, representing the interaction of the model with its distal compartments.
Discretisation	To divide into discrete elements or time periods.
Electrical analogue	An electrical circuit design used to represent a compartment of the circulation, using, for example, ‘voltages’ (pressures), ‘current’ (flow) and resistors. They lack spatial dimensions and are therefore also referred to as dimensionless or ‘*zero-D*’ (0D) models.
In silico	‘Represented or simulated in a computer’, comparable to in vivo and in vitro.
Multi-scale model	A model which integrates models of different length- and or time-scales.
Newtonian and non-Newtonian fluid	As blood is a suspension, non-Newtonian behaviour is particularly important within the capillaries where the size of (solid) blood cells is large relative to vessel calibre, resulting in a non-linear relationship between shear-stress and viscosity. In larger blood vessels Newtonian fluid behaviour is often assumed whereby viscosity is constant, independent of the shear-stress acting on the fluid.
Numerical solution	In more complex models the mathematics becomes too complicated for analytical techniques and numerical techniques are used instead. Rather than generating an exact solution, the result is an approximation, albeit within very close bounds under certain circumstances. Typically, iterative methods are employed to produce a solution to the equations that converges around the true values. Used to resolve complicated, non-linear, transient (time-varying) analyses for example, 3D-CFD models.
Poiseuille equation: 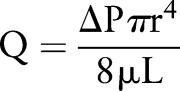 *Rearranged:* 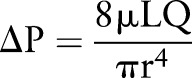	The Poiseuille equation describes blood flow (Q), along a vessel in relation to viscosity (μ), vessel geometry (length (L) and radius (r)) and the driving pressure gradient (ΔP). According to Poiseuille, flow is strongly dependent upon vessel radius (fourth power). Poisuille's equation considers *viscous* (frictional) energy losses.
Segmentation	The process by which relevant structures in medical images are identified, isolated and converted into computer representations.
Windkessel	German for ‘*air-chamber*’. Windkessel models are relatively simple zero-D models used to represent the resistive and compliant properties of the arterial vasculature.
Workflow	A sequence of applications (computational tools) which are executed sequentially to manipulate medical data to build a model and perform computational analyses. Typically this involves medical imaging, segmentation, discretisation, CFD simulation and post-processing, that is, from clinical imaging to results. Sometimes referred to as a *tool-chain*.

CFD, computational fluid dynamics.

For incompressible flows, almost all CFD analyses solve the Navier-Stokes and continuity equations which govern fluid motion. These equations are non-linear, partial differential equations based upon the principle of conservation of mass and momentum. Simplification of these equations yields familiar formulae (eg, those of Bernoulli and Poiseuille); but for complex geometries analytical solutions are not possible, so specialised software applications (CFD solvers) calculate approximate numerical solutions. Non-linearity, due to convective fluid acceleration, makes this challenging, especially in three dimensional (3D) models; so CFD analyses require significant computational power and time.

## CFD model complexity

The applications reviewed in this paper focus on 3D CFD analyses of local regions of the vasculature because this is where promising applications are beginning to translate and impact upon clinical medicine. There is a long history of simplification of the governing equations to lower spatial dimensions. Table 3 summarises the relationship between these approaches and provides clinical examples of their use.

**Table 3:** A summary of the various orders of CFD modelling applied to the cardiovascular system

Model	Figure	CFD solution	Description/examples	Typical solution time^†^
0D	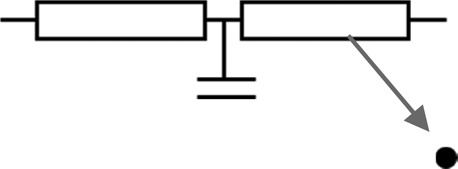 *	No spatial dimension. Physiological variables such as pressure (*P*), flow (*Q*) and resistance (*R*) are assumed spatially uniform within the model, varying only as a function of time (*t*), eg, 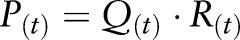 Solved with ordinary differential (0D NS) equations	Lump together distributed physiological systems into a single description. They describe the global behaviour of the modelled segment. The 0D Windkessel model (pictured) is often used to represent the compliant and resistive nature of the arterial circulation. 0D models are frequently used to model components of the cardiovascular system or to improve boundary conditions for 3D models of arterial, ventricular or venous pathophysiology.^5^ ^47^	Immediate solution
1D	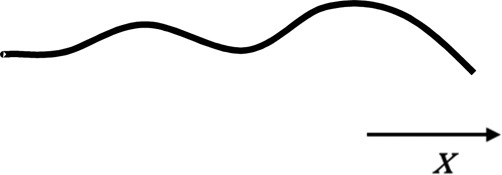	Physiological variables are solved as a function of a single spatial variable, typically length (*x*), eg, 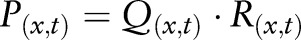 Solved with partial differential (1D NS) equations	Used to represent wave propagation characteristics and wave reflection. 1D models may also be used to provide boundary conditions for higher order models in order to increase refinement of the solution, especially where the effects of wave reflection are significant.^45^ ^46^	S (static) Min (transient)
2D	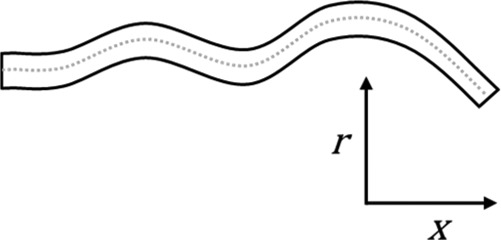	Physiological variables are solved as a function of two spatial variables, typically length and distance from centreline (*r*) eg, 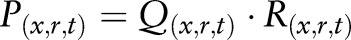 Solved with axisymmetric NS equations	Able to resolve the solution in 2D. Used less often now than previously due to ready availability of improved computer processing and 3D solvers. Examples include the simulation of para-prosthetic valve haemolysis and improvement of the assessment of the proximal flow convergence zone in the clinical evaluation of regurgitant valve disease.^48^	
3D	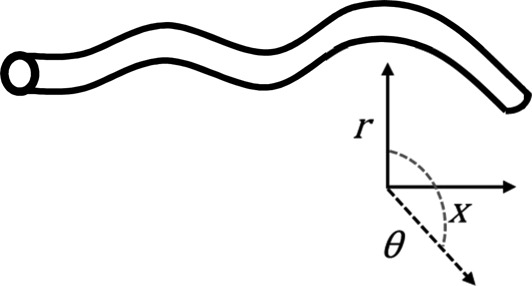	Physiological variables are solved as a function of all three spatial variables, including the angle around the centreline axis (*θ*) eg, 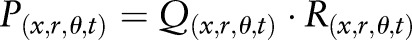 Solved with full 3-D NS equations	Full 3D CFD can resolve the physiological solution in all dimensions including time. Examples are more widely reviewed in the main body of the text.	Order of minutes for steady- state Order of hours or days for transient

*Hydro-electrical analogue diagrams are often used to describe physiological components such as resistance, pressure (voltage), compliance (capacitance), and flow (current).

†Solution times vary according to complexity of the model and the mathematical solution. The times presented are approximate and are based on a model of coronary physiology.^5^

CFD, computational fluid dynamics; NS, Navier-Stokes; 0D, zero dimensional; 1D, one dimensional; 2D, two dimensional; 3D, three dimensional.

2D analyses typically assume symmetry of the solution about the central axis, 1D models capture variation of the solution along the axial direction only, and 0D representations lump the behaviour of vascular regions into a model with no spatial dimensions, hence the term ‘lumped-parameter models'. Due to the breadth of the literature covering application of these techniques to cardiovascular haemodynamics, the interested reader is referred to recent reviews of the state-of-the-art.^45^ ^46^

## Model construction

**Figure 1:**
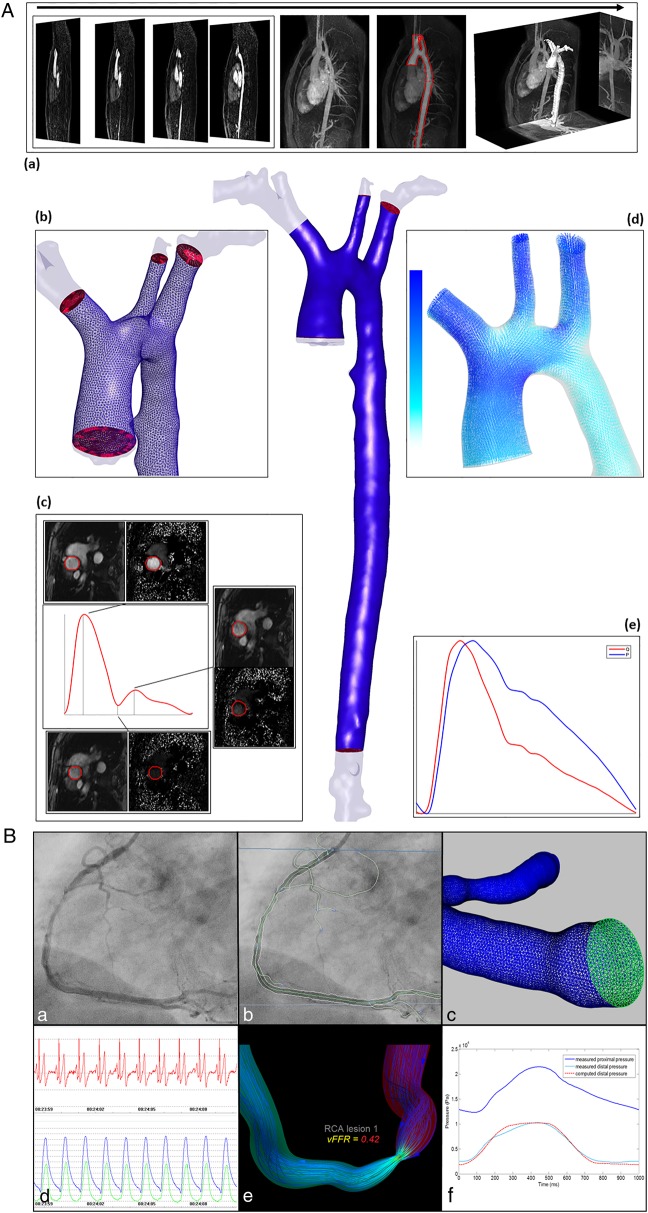
Examples of aortic (A) and coronary (B) in silico computational fluid dynamics (CFD) workflows. (A) The aorta is identified from thoracic MRI (a), segmented and reconstructed (central image). A volumetric mesh is fabricated to fit the patient-specific geometry, shown in detail in panel (b). Accurate flow measurements are extracted from phase-contrast MRI data to inform the boundary conditions applied for CFD simulation, such as the inlet (c). The results are post-processed, details of the flow field are shown in panel (d). 0D models are coupled at the outlets so physiologically feasible flow-pressure relationships are computed at the outlets (e). These can be validated against other measurements, which in a preclinical scenario may be invasive. (B) (and accompanying online video) A coronary angiogram (a) is segmented (b) and reconstructed into a 3D in silico model. A surface and volumetric are fabricated to fit the patient-specific geometry (c). Physiological parameters such as pressure and flow are used to inform the boundary conditions applied for CFD simulation (d). The results (here pressure and flow) are post-processed and useful physiological data are extracted (e). In the preclinical, research setting simulated results are validated against an appropriate standard, for example, invasively measured values (f). (Additional information for video legend): VIRTUheart is an academic project at the University of Sheffield funded by research grants (see virtuheart.com).

CFD model construction and solution can be described in seven stages (figure 1):

1. Clinical imaging

A range of medical imaging modalities can be used, including ultrasound, CT, MRI and X-ray angiography. Imaging must provide sufficient anatomical and physiological detail, in an appropriate format and quality, to enable segmentation and data extraction.^49^

- Segmentation and reconstruction

Segmentation methods convert medical images to in silico geometries which define the physical bounds of the model region of interest. If images are acquired over a cardiac cycle, anatomical motion can be tracked over segmented regions.^50^ ^51^

- Discretisation

Spatial discretisation, or ‘meshing’, divides the geometry into a number of discrete volumetric elements or cells. Temporal discretisation divides the solution into discrete time steps. The accuracy and numerical stability of the analysis are influenced by both spatial and temporal refinement. The fabrication of the mesh, and the level of mesh refinement, are influenced by case- and context-specific factors. The mesh and timestep (ie, spatio-temporal discretisation) must be refined enough to capture the important haemodynamic behaviour of the modelled compartment (the final solution should be independent of mesh parameters), but without excessive refinement because this impacts negatively on computational resource and solution time (see online supplementary table S1).

- Boundary conditions

Because it is impossible to discretise the entire cardiovascular system, the region to be analysed will have at least one inlet and one outlet. To enable CFD analysis, the physiological conditions at the wall and inlet/outlet boundaries must be specified. Boundary conditions are a set of applied physiological parameters (which may vary over time) that define the physical conditions at the inlets, outlets and walls. They may be based on patient-specific data, population data, physical models or assumptions.^39^

- Simulation

A computer file defining the physical parameters of the model is written. In addition to the geometric, discretisation and boundary data, this file must define properties including: blood density and viscosity (ie, the fluid model), the initial conditions of the system (eg, whether the fluid is initially static or moving), time discretisation information (time step size and numerical approximation schemes), and the desired output data (eg, number of cardiac cycles to be simulated). This information allows the CFD solver to solve the Navier-Stokes and continuity equations, proceeding incrementally towards a final solution (‘convergence’). A typical 3D cardiovascular simulation involves >1 million elements run over several cardiac cycles, each divided into hundreds or thousands of individual time-steps. Millions of non-linear partial differential equations are solved simultaneously, and repeatedly, over all elements, at all time-steps. 3D CFD modelling is therefore time-consuming and computationally demanding.

- Post-processing

Typically, the CFD solver produces the pressure and velocity field over all elements at each time-step. Only a small proportion of these data are of interest to the operator, so some post-processing is required to extract and display relevant data.^18^

- Validation

It is important that modelled results are validated against an acceptable standard. Commonly, this involves comparison with either values measured within an in vitro phantom or acquired during in vivo assessment.^4^ Validation generates confidence in the accuracy and reliability of a CFD model.

## The workflow

Collectively, the steps outlined above are known as a *workflow* or *toolchain* (see figure 1 and online supplementary video). Although there are many specialised software applications facilitating the construction and operation of CFD-based workflows, considerable skill and experience are required at each stage (especially steps 3–5) to ensure reliability of results.^49^

## Advanced boundary conditions

**Figure 2:**
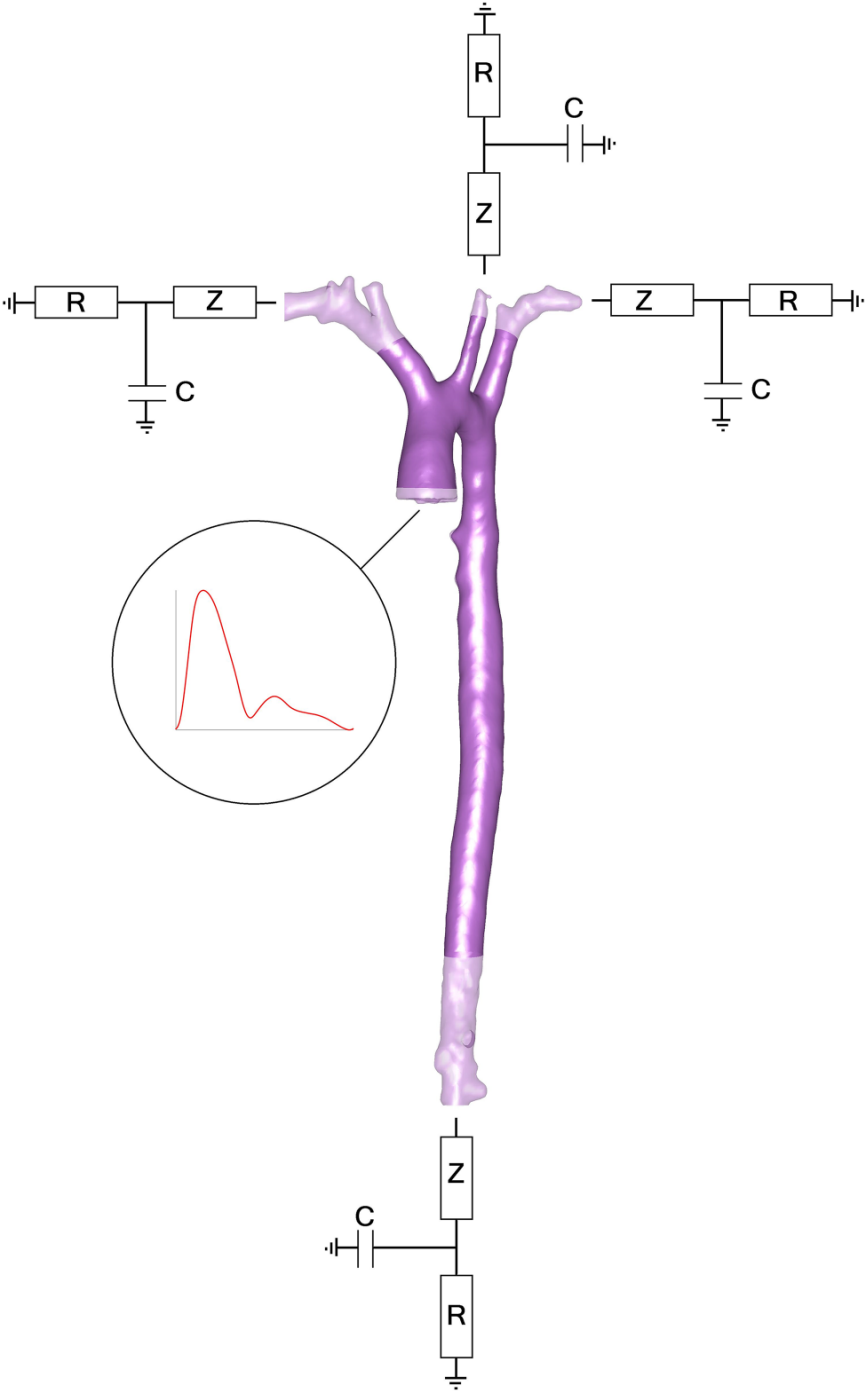
A patient-specific 3D computational fluid dynamics model of an aorta. Patient-specific pressure is the proximal boundary condition. Each outlet (distal boundary) is coupled to a zero-dimensional model. The zero-dimensional models represent the impedance (Z), resistance (R) and compliance/capacitance (C) of the circulation distal to the boundaries. Output data from the 3D domain provide input to the 0D model and vice versa. The algebraically coded 0D models compute parameters which are returned back to dynamically inform the 3D simulation. An alternative would be to couple a 1D wave transmission model at the outlets which may provide higher fidelity simulation results, especially in the aorta where the physiology is influenced by wave reflections.

Rather than specifying pressure or flow at a boundary, an additional, lower-order model may be coupled to the 3D solver to generate more realistic conditions proximal and distal to the simulation domain (see table 1 and figure 2). This method of modelling is efficient, because it allows detailed analysis in the 3D region without wasting high temporal and spatial refinement on regions beyond this. In some cases the model representing the distal boundary also provides proximal boundary condition. These closed-loop models, or *system models,* require very careful tuning.^52^

## Assumptions

Many CFD models assume that the segmented region has rigid walls. Although untrue in the cardiovascular system, this approximation is acceptable for some applications.^53^ ^54^ Vessel compliance allows blood to be stored during systole and released during diastole. At the system level this results in a finite speed of pressure wave transmission and tends to reduce the peak pressures associated with the inertial acceleration of the blood. Compliance tends to reduce shear stresses because the vessels are slightly larger when peak flow occurs. It is possible to model deformation of the wall, due to cardiac and respiratory variation, in response to change in pressure using fluid-solid interaction (FSI) models. These are far more complex to solve, boundary conditions are a challenge, and many wall parameters remain unknown, which increases the number of assumptions. Furthermore, it is yet to be established for which applications a full FSI approach improves accuracy. An example of the increased computational cost of FSI is reported by Brown *et al*^54^ where 3D transient (time-varying) analysis of the aorta required 145 h (FSI) compared with 6.6 h (CFD). An alternative is to impose wall movement derived from imaging data (eg, gated MRI). There are exciting developments in the use of data assimilation techniques in which sparse clinical data, for example, from 4D imaging, are integrated with the analysis process so that material properties of tissues in individual patients are recovered as the simulation progresses.^55^ In biomedical workflows it is assumed the boundaries of the fluid geometry are smooth, yet medical images may not generate smooth surfaces due to poor resolution or imaging artefacts. Instead, structures may be smoothed in silico after segmentation. Typically, cardiovascular simulations assume blood behaves as an incompressible fluid. Although blood exhibits non-Newtonian behaviour (see glossary in table 2), which must be simulated for flow in small capillaries, in larger vessels these effects are often neglected and a Newtonian fluid model is assumed.

## Benefits of cardiovascular CFD modelling

**Figure 3:**
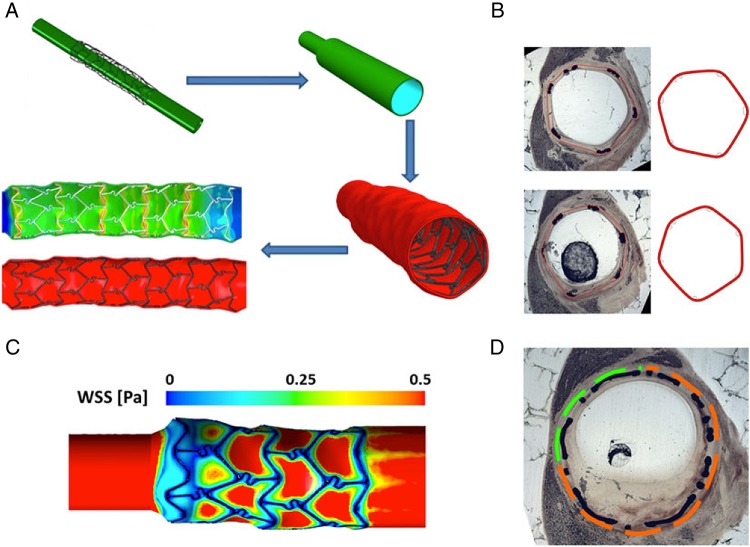
A computational fluid dynamics (CFD) model demonstrating the correlation between wall shear stress (WSS) and restenosis in coronary artery disease. (A) Structural modelling of stent insertion in porcine coronary arteries reconstructed from micro-CT, and stent–artery coupling obtained after arterial recoil. (B) Comparison between the in vivo histological images (left) and corresponding sections from the structural simulation (right) demonstrating excellent agreement. (C) Results of the CFD simulations in terms of the spatial distribution of WSS magnitude over the arterial wall. (D) The correlation between areas characterised by low WSS (orange lines) and in-stent restenosis after 14 days. The CFD simulation of WSS has identified areas of reduced shear and restenosis with excellent agreement. Figure reproduced from Morlacchi *et al*^26^ with kind permission from Springer Science and Business Media.

CFD modelling enables investigation of pressure and flow fields at a temporal and spatial resolution unachievable by any clinical methodology. Post-processing provides additional data, generating new insights into physiology and disease processes. For example, it is difficult, and invasive, to measure arterial wall shear stress (WSS), a key factor in the development of atherosclerosis and in-stent restenosis, whereas CFD models can compute WSS and map its spatial distribution.^20^ ^21^ Such work has established the link between haemodynamic disturbance and atherogenesis and has explained the preferential deposition of atherosclerotic plaque at arterial bends and bifurcation regions.^22^ CFD modelling has been central to our current understanding of the effects of WSS on endothelial homeostasis: laminar, non-disturbed blood flow is associated with increased WSS which inhibits unnecessary endothelial cell activation; whereas turbulent or disturbed blood flow reduces WSS which stimulates adverse vessel remodelling. A complex series of WSS-related signalling pathways and interactions underlie this phenomenon. Before these pathways can be exploited to generate anti-atherosclerotic therapies their complexity needs to be better understood. Integrated multiscale CFD models provide a powerful tool to combine analysis of fluid mechanics and cellular response.^23^ Recently, the effect of disturbed WSS in stented vessels has been modelled to investigate the influence on both endothelial function and neointimal hyperplasia, which preferentially accumulates at regions of low and disturbed WSS (figure 3).^26^ Such models can be used to develop stents which minimise the risk of in-stent restenosis and thrombosis.^24^ ^25^

### Device design

In silico methods allow rapid prototyping, with reduced risk to humans, so a priority for the medical devices industry is to replace expensive and time-consuming in vivo and in vitro experimentation with in silico testing. In recognition of this, the US Food and Drug Administration (FDA) issued draft guidance in 2014 on the use of modelling to support regulatory submissions.^56^ An example is the significant role which CFD plays in design optimisation of mechanical heart valves.^11^ While comparison of major flow features captured in vitro with predictions from CFD shows good agreement, simulation delivers 3D information at much higher resolution within critical regions (eg, the hinges) than flow visualisation, giving invaluable insight into design-related thrombogenic potential.^10^ Comprehensive simulation of heart valve mechanics, including the separation of the upstream and downstream fluid regions at closure and the structural instability and snap-through dynamics of tissue valves, remain computationally challenging but achievable.^9^ ^13^

CFD has been used in the optimisation of several commercial ventricular assist devices (VADs), investigating potential thrombogenicity by highlighting design-related regions of stasis and device characteristics resulting in high shear stress.^41^ A tool for optimising thromboresistance has been recently demonstrated in a comparative study of two continuous flow VADs, which combines experimental and numerical simulation.^40^ ^57^ Numerical models can also contribute to the process of VAD implantation, informing the choice of catheter implantation site.^39^

In the context of stent design, the greatest emphasis has been on modelling the mechanical integrity of the stent structure during and after deployment. However, CFD provides a valuable tool to assess the resulting haemodynamics within the stented lesion.^26^ This, in turn, has been associated with the biological response of the vessel wall and the development of restenosis.^27^

### Diagnostic tools and personalised medicine

Computing intravascular physiology, with the aim of minimising invasive instrumentation, is of major interest. A prime example is fractional flow reserve (FFR), an index of physiological (coronary atherosclerosis) lesion significance, measured with a pressure-sensitive angioplasty guidewire. FFR-guided therapy improves patient outcomes, reduces stent insertions, and reduces costs, yet it is used in <10% of cases due to a host of procedural and operator related factors.^58^ Several groups propose models for computing ‘virtual’ FFR from angiography to provide the benefits of physiological assessment without the practical drawbacks which limit the invasive technique (see online supplementary figure S1).^5^ ^6^ ^7^ ^59^ The VIRTU-1 trial demonstrated the effectiveness of CFD-derived FFR using invasive angiography (diagnostic accuracy 97% vs invasive FFR).^5^ More recently, the HeartFlow-NXT trial demonstrated the performance of virtual FFR using CT coronary angiography (sensitivity 86%, specificity 79% vs invasive FFR).^6^ Both models offer a less invasive approach, neither requires hyperaemic flow induction nor the passage of an intracoronary wire, and taken together could offer the benefits of FFR to all patients being assessed for coronary artery disease (CAD). _CT_FFR (HeartFlow Inc) is now FDA approved for use as a class II Coronary Physiologic Simulation Software Device.^60^

Recent in vitro work combining CFD, colour Doppler and simulated Doppler images demonstrates the potential to reduce Doppler inter-user variability and inform interpretation of complex regurgitant flow fields in valvular heart disease.^61^ In silico models of the right heart and pulmonary arteries using phase-contrast MRI capture anatomical and flow velocity data to simulate pulmonary artery physiology.^62^ It is hoped that these models will soon deliver a diagnosis of pulmonary hypertension without invasive catheterisation.^31^ Added value may come from wave analysis of these models to discriminate between the key aetiological sub-groups of pulmonary hypertension.^30^

Treatment decisions are often based upon the ‘gold standard’ of randomised controlled trials. Everyday clinical practice, however, requires tailored treatment for individual patients. CFD modelling offers a patient-specific approach to management, in which an individual's unique anatomy and physiology are used to define the model. The impact of alternative interventional strategies can be compared and a personalised, optimised strategy selected.^63^ Patient-specific modelling will not diminish the need for clinical trials but will allow the delivery of truly objective, personalised management on a wide scale.

**Figure 4:**
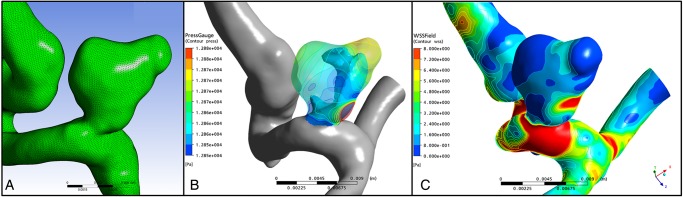
Computational fluid dynamics (CFD) model of an intracranial berry aneurysm from the @neurist project. Panel (A) demonstrates the reconstructed surface mesh. Panels (B) and (C) demonstrate the CFD simulated pressure (B) and wall shear stress (C) acting upon the aneurysm wall, which may be useful in predicting risk of rupture on a patient-specific basis.

**Figure 5:**
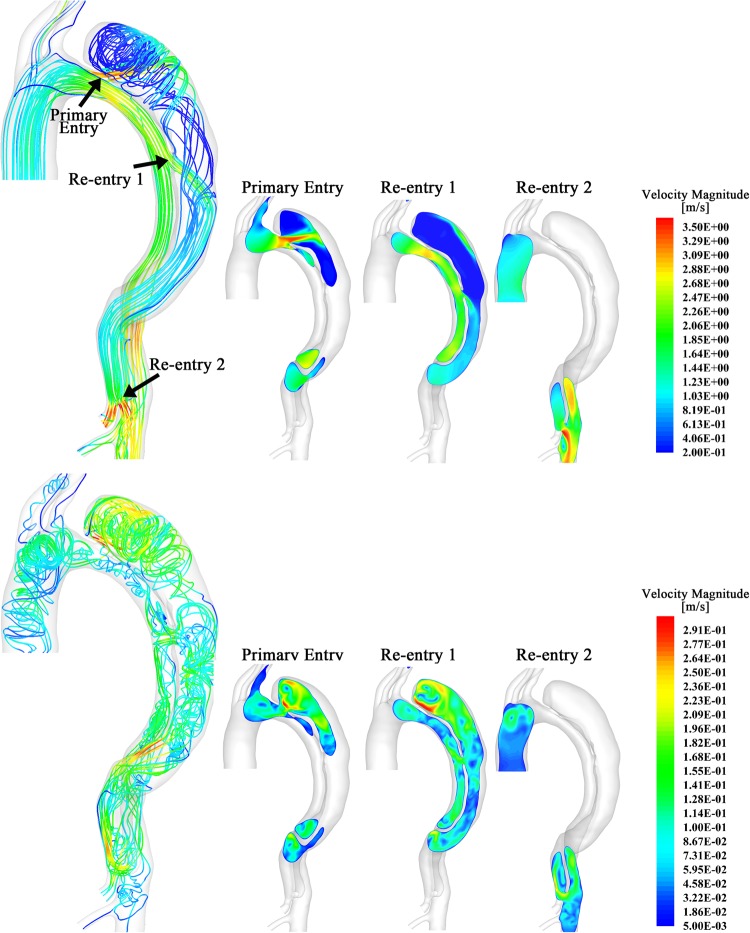
Segmentation, reconstruction and 3D simulation of a chronic type B aortic dissection with true and false lumen in systole (top row) and diastole (bottom row). The primary entry point (top arrow) is close to the left subclavian artery. Two more communications (‘re-entries’) are seen distally. Computational fluid dynamics simulation allows the flow through each re-entry point to be studied separately in order to predict response to intervention. During systole simulation demonstrates high blood flow velocity through the primary entry point. However, simulation predicts significant flow through the first re-entry point in systole, and even higher during diastole, thus demonstrating that closure of the primary entry point alone will not be sufficient to induce false lumen thrombosis and avoid further expansion. Reproduced with permission from Chen *et al*, 2013.^18^

This approach is exemplified in aortic aneurysm management, where current simplistic guidelines use aneurysm diameter as the arbiter of treatment. The @neurIST project addresses this, and incorporates patient-specific anatomic, genomic and demographic data, with simulated flow patterns (figure 4), to calculate the risk of rupture.^64^ The optimal treatment of aortic type B dissections remains controversial. Thoracic endovascular aortic repair (TEVAR) carries a risk of spinal ischaemia, and multiple communicating channels between true and false lumens confer a risk of proximal rupture if only the primary entry is closed. Therefore, graft length is balanced against the risk of paraplegia. In this context, CFD modelling provides individualised risk stratification and optimised treatment delivery (figure 5).^65^ ^18^ Similar simulations are useful in the context of abdominal aortic aneurysm.^66^ ^67^

## Full system models: the Virtual Physiological Human

There is increasing interest in integrating multiple physiological models into comprehensive system models to simulate the impact of various conditions, pathologies and treatments across multiple organ systems. While this is ambitious, large, international, collaborative research projects under the umbrella of the Virtual Physiological Human (VPH) reflect the seriousness, legitimacy and rationale underlying this long-term vision.^68^ Full system models offer the possibility of understanding, holistically, the impact of cardiovascular disease upon individual patients.

## In silico trials

In silico techniques can simulate measures of safety, accuracy, and efficacy of interventions, both pharmacological and mechanical, in large cohorts of virtual patient models which represent naturally occurring physiological and pathological variability. This minimises the time, cost, and risk associated with clinical trials. The Avicenna project leverages significant commercial interest and engagement to develop a ‘roadmap’ describing the route by which multi-scale in silico techniques can achieve this.^69^

## Challenges and limitations

CFD models in medicine have traditionally been used by two user groups: industrial medical device developers, in rapid, low-cost, device prototyping; and academics, to investigate cardiovascular physiology and compute parameters that cannot otherwise be obtained. Both groups construct models which are typically complex, involve multiple finely-tuned geometric, haemodynamic and material parameters and require long computation times. In contrast, clinicians are a third, emerging user group, who require rapid results with adequate accuracy.

Model accuracy is determined by model design and quality of input data. For CFD applications it is unclear how detailed the clinical data needs to be in terms of *geometry* (segmented from medical images) and *parameterisation* (variability described by the model and the tuning of patient-specific boundary conditions). Continuing improvements in imaging, image-registration and segmentation algorithms will augment accuracy.^50^ Model parameterisation is more challenging, because it requires detailed knowledge of physiological metrics in the proximal and distal circulations which may be inaccessible and variable in health and in disease, for example, microcirculatory resistance is a major determinant of coronary blood flow. Further understanding of the relative importance of physiological parameters is required to determine those which are most influential, and those which can be assumed or averaged.^70^ This allows unnecessary model complexity to be simplified, balancing computing speed against accuracy.^54^ A second challenge is the development of relevant industry standards. In the European Union and USA, diagnostic software is regulated via CE marking and FDA directives, respectively; but there are no industry standards governing accuracy, reliability or validation. The FDA is addressing this through benchmarking initiatives, in the same way that aviation authorities adopted computer-aided design over traditional physical testing.^71^ Third, large volumes of clinical data, of value for model development and validation, are stored in hospital systems. Access is variable and restrictive; although the VPH-Share project demonstrates how anonymised patient-specific data and in silico models can be shared securely.^72^ Access to such data supports model validation against long-term outcomes which may expedite clinical translation. Fourth, CFD modelling can be perceived as a ‘disruptive technology’, and a threat, especially by manufacturers of traditional hardware. Finally, the next generation of doctors will require training in in silico systems, and understanding their principles, methodologies and limitations. Several initiatives are attempting to address this, but in the longer term, in silico medicine may gain prominence in medical curricula.^68^ ^73^ ^74^

## Future directions

In the context of device development, the major computational modelling and technological challenges have already been addressed and the benefits of simulation are recognised by the regulatory authorities. Sustained investment will enable engineers to continue model refinement and the development of novel applications, specifically targeting increases in precision, personalisation and speed. Beyond technological development, and before these tools become established in routine clinical practice, the most immediate need is to demonstrate equivalence of in silico results relative to invasive measurements through observational trials. Beyond this, efficacy must be demonstrated in large multicentre clinical trials. It is clear that these techniques have the potential to change clinical practice. The beneficiaries will be patients, clinicians and healthcare providers.

## Supplementary Material

Web figure

Web table

Web video
