# Supplementary material for: Computational fluid dynamics modelling in cardiovascular medicine
Source: Heart. 2015 Oct 28;102(1):18–28. doi: 10.1136/heartjnl-2015-308044 (PMC4717410; doi:10.1136/heartjnl-2015-308044)

Supplementary Figure 1 (online only). An example of a 3-D CFD model of coronary arterial physiology. A severe right coronary artery lesion is (a) segmented and (b) reconstructed into a 3-D anatomical computer model. CFD analysis simulates pressure and flow and the results can be seen in (b) and (c). Simulation is transient (dynamic) in order to capture the acceleration and deceleration of pulsatile blood flow. The pressure and flow results are demonstrated at a single point in the cardiac cycle. The predicted 'virtual' FFR (vFFR) was computed from the simulation results over the entire cardiac cycle. The predicted FFR was 0.38 and the corresponding FFR measured in the catheter laboratory was 0.40.

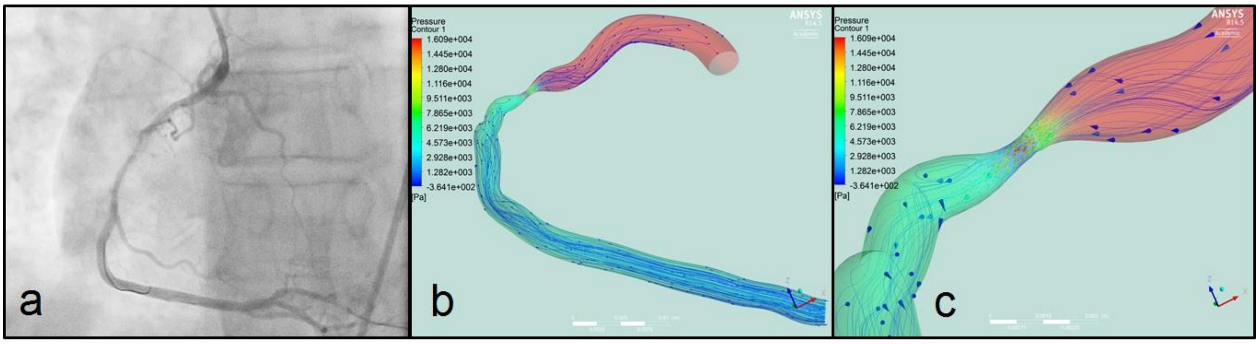

Supplement: Web figure [file heartjnl-2015-308044-s1.pdf]
