# Supplementary material for: Computational fluid dynamics modelling in cardiovascular medicine
Source: Heart. 2015 Oct 28;102(1):18–28. doi: 10.1136/heartjnl-2015-308044 (PMC4717410; doi:10.1136/heartjnl-2015-308044)
Supplement: Web table [file heartjnl-2015-308044-s2.pdf]

Supplementary Table 1 (for online appendix only). *The relationship between mesh element size, solution and solution time for a typical 3-D transient CFD cardiovascular model*

	Start	Varying global mesh element size		Varying surface volume element size			
Alteration	Reference	75%	125%	75%	56.25%	125%	156.25%
GMES (mm)	0.25	0.1875	0.3125	0.25	0.25	0.25	0.25
SVES (mm)	0.125	0.125	0.125	0.09375	0.070313	0.15625	0.195313
Tetrahedral elements (n)	309550	1052602	310099	674827	1381938	557375	294336
Prisms elements (n)	9.97×10^5	10.0×10^5	9.96×10^5	1.77×10^6	3.15×10^6	6.40×10^5	4.10×10^5
Total elements (n)	1.3×10^6	2.1×10^6	1.3×10^6	2.4×10^6	4.5×10^6	1.2×10^6	0.7×10^6
Solution pressure (KPa)	2.603	2.618	2.608	2.639	2.636	2.598	2.507
Solution time (mins)	5882	8400	4680	9555	11670	2399	1230
Solution time (days)	4.08	5.83	3.25	6.64	8.10	1.67	0.85

Data presented are for a model of coronary arterial physiology [5]
